# Understanding the role of ursodeoxycholic acid and gut microbiome in non-alcoholic fatty liver disease: current evidence and perspectives

**DOI:** 10.3389/fphar.2024.1371574

**Published:** 2024-03-21

**Authors:** Qingyi Mao, Beibei Lin, Wenluo Zhang, Yu Zhang, Yu Zhang, Qian Cao, Mengque Xu

**Affiliations:** ^1^ Department of Gastroenterology, Sir Run Run Shaw Hospital, College of Medicine, Zhejiang University, Hangzhou, China; ^2^ Inflammatory Bowel Disease Center, Sir Run Run Shaw Hospital, College of Medicine, Zhejiang University, Hangzhou, China; ^3^ Institute of Gastroenterology, Zhejiang University, Hangzhou, China

**Keywords:** ursodeoxycholic acid, gut microbiome, bile acid receptors, non-alcoholic fatty liver disease, bile acid–gut microbiome axis

## Abstract

Non-alcoholic fatty liver disease (NAFLD) is the most common chronic liver disease, resulting in a huge medical burden worldwide. Accumulating evidence suggests that the gut microbiome and bile acids play pivotal roles during the development of NAFLD. Patients with NAFLD exhibit unique signatures of the intestinal microbiome marked by the priority of Gram-negative bacteria, decreased ratio of *Firmicutes/Bacteroidetes* (F/B), and increased *Prevotella* and *Lachnospiraceae*. The intestinal microbiota is involved in the metabolism of bile acids. Ursodeoxycholic acid (UDCA) is a key determinant in maintaining the dynamic communication between the host and gut microbiota. It generally shows surprising therapeutic potential in NAFLD with several mechanisms, such as improving cellular autophagy, apoptosis, and mitochondrial functions. This action is based on its direct or indirect effect, targeting the farnesoid X receptor (FXR) and various other nuclear receptors. This review aims to discuss the current studies on the involvement of the microbiome–UDCA interface in NAFLD therapy and provide prospective insights into future preventative and therapeutic approaches for NAFLD.

## 1 Introduction

Non-alcoholic fatty liver disease (NAFLD) is defined as the presence of ≥5% hepatic steatosis, which is not secondary to specific reasons such as significant alcohol consumption and drug damage. NAFLD is caused by metabolic dysfunction, being overweight or obese, and type 2 diabetes mellitus. Encompassing a broad spectrum of clinical phenotypes, NAFLD ranges from simple steatosis to non-alcoholic steatohepatitis (NASH), advanced liver fibrosis, cirrhosis, and hepatocellular carcinoma (Chalasani et al., 2018). The prevalence of NAFLD is estimated to be 20%–30% worldwide and is constantly increasing due to its association with obesity, diabetes, and metabolic syndrome (Younossi et al., 2016). Currently considered the second leading indication for liver transplantation, with numbers further increasing, NAFLD has become a heavy global burden that requires immediate attention (Younossi et al., 2018). The etiology of NAFLD is complex, including dietary and environmental factors that potentially lead to apoptosis, metabolism abnormalities, oxidative stress, and inflammation in the liver. In addition, the effect of patatin-like phospholipase domain-containing 3 (PNPLA3), transmembrane 6 superfamily member 2 protein (TM6SF2), and membrane-bound O-acyltransferase domain-containing 7 (MBOAT7) gene polymorphism provides convincing evidence for elucidating the genetic susceptibility to NAFLD (Xia et al., 2022). The application of Mendelian randomization has helped researchers discover more gene targets, like lipoprotein lipase (LPL) (Li et al., 2023). Recently, a novel hypothesis of “Multiple parallel hits” has been proposed, which suggests that gene polymorphism, lipotoxicity, and intestinal microbiome together contribute to the development of NAFLD (Tilg et al., 2021). However, the precise molecular network underlying NAFLD remains unclear, which is crucial for identifying novel and effective therapeutic targets to prevent or at least reduce the growing burden of liver transplantation.

Many studies have shed light on the indispensable role of the gut microbiome in the acceleration of NAFLD progression (Abenavoli et al., 2022). Mechanisms regarding the gut microbiome in NAFLD involve increased permeability of the intestinal barrier and perturbation of various bacterial metabolites, like bile acids and ethanol (Jiao et al., 2018; He et al., 2022). The gut–liver axis also occupies an important position in both mechanisms by providing a shortcut (Milosevic et al., 2019). Furthermore, microbiome features have been used to develop a new non-invasive risk assessment tool with a random forest machine learning model in NAFLD patients (Elsayed et al., 2022; Leung et al., 2022). It can provide potential new therapeutic strategies like probiotics and fecal microbiota transplantation (FMT) to attenuate chronic liver diseases (Scorletti et al., 2020; Xue et al., 2022). Of note, bile acids play a critical role in regulating metabolism and hepatic pathophysiology. They are initially synthesized as primary bile acids (PBAs) in the liver and then transformed into secondary bile acids (SBAs) by the gut microbiome. Although the microbiome is a prerequisite for SBA production, specific bile acids possess the capability to reciprocally modulate gut microbiome growth (Winston et al., 2020). In addition, it has been suggested that NAFLD is characterized by augmented synthesis of bile acids independent of downregulated bile acid signaling (Jiao et al., 2018). The modulation of bile acid profiles also represents a key step in ameliorating NAFLD (Jiao et al., 2018; Sun et al., 2019; Juarez-Fernandez et al., 2021). Indeed, modifications to the bile acid pool may serve as a new interventional target for NAFLD, as certified in both animal and human experiments (Kim et al., 2019; Gillard et al., 2022).

Ursodeoxycholic acid (UDCA) a secondary bile acid with the highest hydrophilicity and lowest toxicity. With anti-inflammatory, antioxidant, and anti-fibrotic properties, UDCA has been approved as the first-line drug for treating cholestatic liver diseases, such as primary biliary cholangitis. However, the evidence on its efficacy for NAFLD therapy is controversial. Studies have shown that UDCA alleviated hepatic steatosis and modulated inflammation and fibrosis (Marchiano et al., 2022; Xie et al., 2022; Marchianò et al., 2023). Despite its limited presence in the bile pool (only 1%–3%), UDCA interacts closely with the gut microbiome and can therefore directly or indirectly influence the progression of NAFLD. It warrants greater attention for its potential as a therapy for NAFLD.

**FIGURE 1:**
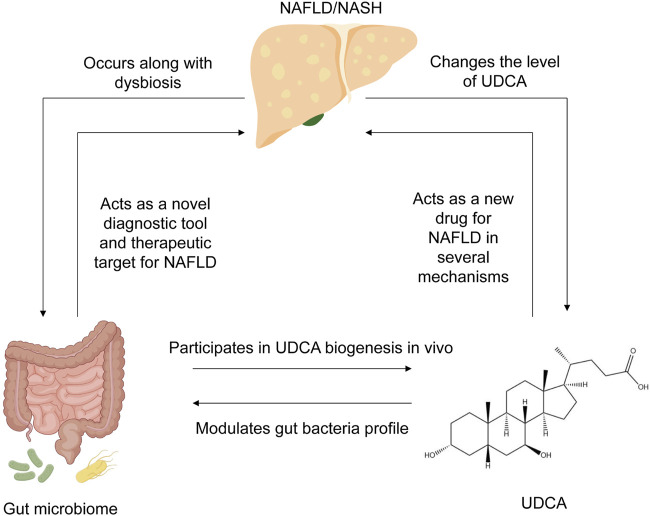
Summary of the interplay between NAFLD, UDCA, and gut microbiome. NAFLD occurs along with dysbiosis in the human gut, changes the level of UDCA, and alters bile acid composition. In turn, the gut microbiome has the potential to act as a novel diagnostic tool for NAFLD diagnosis and evaluation, as well as an effective therapeutic target for NAFLD therapy. UDCA, produced by the gut microbiome *in vivo*, can also remodel the bacterial community in the human gut. NAFLD, non-alcoholic fatty liver disease; UDCA, ursodeoxycholic acid.

In this review, we summarized the interaction between the gut microbiome, UDCA, and NAFLD, as well as the underlying mechanisms as revealed by recent research studies (Figure 1). The role of the gut microbiome and UDCA in liver immune response and inflammation will be discussed, with implications for development of preventive strategies, new therapeutics, and potential diagnostic tools for clinical applications in NAFLD.

## 2 Gut microbiome signatures and NAFLD

It has been clearly indicated that NAFLD patients harbored an obviously lower diversity and richness of the gut microbiome than healthy controls (Caussy et al., 2019; Schwimmer et al., 2019; Hullar et al., 2021). Gram-negative (G^−^) bacteria are prevalent in both obese and non-obese NAFLD patients (Wang et al., 2016; Schwimmer et al., 2019), which is represented by the phylum Proteobacteria as well as the genera *Prevotella* and *Bacteroides*. Critically, the genus *Escherichia Shigella* is sharply increased in the NAFLD group, especially in patients with significant fibrosis (Shen et al., 2017). Alternatively, two pro-inflammatory responses associated with G^−^ bacteria, including genes for lipopolysaccharide biosynthesis in NASH and genes for flagellar assembly in moderate-to-severe fibrosis, are enriched in the NAFLD metagenome (Schwimmer et al., 2019). Collectively, dysbiosis that might contribute to pathogenesis of NAFLD is normally presented as endotoxemia, gut barrier leakiness, and subsequent inflammatory gene overexpression in the liver.

At the phylum level, *Bacteroidetes* and *Firmicutes* are the two dominant groups in the microbial community, with the prevalence accounting for over 90% in the human gut (Nkosi et al., 2022). Changes in the ratio of *Firmicutes/Bacteroidetes* (F/B) are considered an index of dysbiosis, whereas research studies regarding the changes in the F/B ratio had inconsistent conclusions. *Firmicutes* and *Bacteroidetes* were previously proposed as “fatty bacteria” and “lean bacteria,” respectively (Zhao, 2013); therefore, it could be speculated that high-fat diet-induced NAFLD theoretically results in the increased proportion of F/B (Carino et al., 2019). In most animal experiments, the F/B ratio followed the predicted tendency in NAFLD models and was restored along with the improvement in steatosis indicators (Porras et al., 2017; Gao et al., 2021; Yu et al., 2021; Romualdo et al., 2022). Both high-fat diet and Western diet-induced mouse NAFLD models showed similar results in the gut microbiome at the phylum level (the detailed information is displayed in Table 1). Interestingly, NAFLD and NASH patients showed different results, such as a higher abundance of *Bacteroides* and a lower abundance of *Firmicutes* (Wang et al., 2016), or no significant difference was observed in the F/B ratio between patients and controls (Tsai et al., 2020). The F/B ratio had contradictory results as it was highly affected by different methods of sequencing (different primer sets, shotgun vs. 16S, etc.), so the overall application, therefore, needed more validation. As predominant members of *Firmicutes*, the two families *Lachnospiraceae* and *Ruminococcaceae* are known as beneficial bacteria that promote human health. *Ruminococcaceae* exerts a protective influence on hepatic histopathology by producing SCFA and 7-α dehydrogenation. The *Ruminococcus* family is less abundant in NAFLD patients than in healthy samples (Tsai et al., 2020; Juárez-Fernández et al., 2023). On the contrary, however, some researchers previously reported that a high abundance of *Ruminococcus* was associated with advanced fibrosis (Boursier et al., 2016). The enrichment of *Lachnospiraceae* and *Blautia* demonstrated a growing tendency in the NAFLD rodent model and NASH patients. This may be attributed to the neutralization of impairment caused by pathogens and the effort to recover the subtle homeostasis during NAFLD progression (Shen et al., 2017). Another suggestion is that *Lachnospiraceae* expresses key bile acid-metabolizing enzymes required for deoxycholic acid (DCA) and downstream metabolite synthesis, while patients with significant liver fibrosis tend to have higher serum DCA levels (Smirnova et al., 2022).

**TABLE 1:** Comparison of the microbiome in control groups vs. different mouse NAFLD models.

Modeling method	Phylum	Family	Genus
Western diet (Yu et al., 2021; Romualdo et al., 2022)	↑F/B ratio, *Firmicutes*, and *Proteobacteria*	—	*↑Helicobacter, KE159600_g*, *Mucispirillum*, *Pseudoflavonifractor*, *Clostridium_g21*, *and Faecalibaculum*
	↓*Bacteroidetes* and *Fusobacteria*		*↓Lactobacillus and PAC000664_g*
High-fat diet (Porras et al., 2017; Gao et al., 2021)	↑*Firmicutes* and *Proteobacteria* and F/B ratio	*↑Veillonellaceae*, *Desulfovibrionaceae*, *Erysipelotrichaceae*, *Enterococcaceae*, *Mogibacteriaceae*, *and Alcaligenaceae*	*↓Akkermansia*
		*↓Turicibacteraceae*, *Dehalobacteriaceae*, *F16*, and *Lactobacillaceae*	*↑Helicobacter*

Abbreviations: NAFLD, non-alcoholic fatty liver disease; F/B, *Firmicutes/Bacteroidetes*.

Studies demonstrated that patients with different stages of NAFLD had their own gut microbial characteristics, which are presented in Table 2. Patients with early-stage NAFLD exhibited a growing abundance of *Firmicutes*; however, patients with severe NAFLD lesions tended to have a lower abundance of *Firmicutes* (Loomba et al., 2017). Although belonging to *Firmicutes*, *Streptoccocus* was found to be enriched in NAFLD with cirrhosis and hepatocellular carcinoma (Caussy et al., 2019; Ponziani et al., 2019). The abundance of G^−^ bacteria, including *Enterobacteriaceae*, *Proteobacteria*, and *Lachnospiraceae* families, represented an increasing trend from simple hepatic steatosis progressing to fibrosis (Loomba et al., 2017; Shen et al., 2017; Caussy et al., 2019; Schwimmer et al., 2019). These bacteria profiles are detrimental to liver health, and the abundance increases as NAFLD progresses, thereby establishing a vicious circle. To assess the severity of NAFLD more accurately, NAFLD patients can be stratified based on multivariate analysis according to the abundance of *Bacteroides* and *Ruminococcus* (Boursier et al., 2016). However, further research is needed to quantify microbiome features as a model using a larger amount of data, which could be a novel method for diagnosing and predicting the prognosis of NAFLD.

**TABLE 2:** Microbiome signatures in patients with NAFLD.

Reference	Population	Lab technique	Microbiome characteristic
Boursier et al. (2016)	57 biopsy-proven NAFLD patients (France)	Fecal microbiome: 16S ribosomal RNA gene sequencing	Patients with NASH and F≥2: ↑*Bacteroides*
			Patients with F≥2: ↑*Ruminococcus*
Wang et al. (2016)	43 NAFLD and 83 healthy controls (China)	Fecal microbiome: 454 pyrosequencing of the 16S ribosomal RNA V3 region	Patients with NAFLD: lower diversity ↓F/B ratio
			↑G negative bacteria
			↓*Clostridia*
			↓*Lachnospiraceae*
			↓*Ruminococcaceae*
Shen et al. (2017)	25 NAFLD patients and 22 healthy subjects (China)	Fecal microbiome: 16S rDNA amplicon sequencing	Patients with NAFLD
			↑*Streptococcus*
			↑*Escherichia_Shigella*
			↑*Lachnospiraceae_Incertae_Sedis*
			↑*Blautia* patients with NASH or F ≥2
			↑either genus *Blautia* (*Lachnospiraceae* family) or genus
			*Escherichia_Shigella* (*Enterobacteriaceae* family)
Rau et al. (2018)	32 NAFLD patients and 27 healthy controls (Germany)	Fecal microbiome: 16S ribosomal RNA gene sequencing	Patients with NASH
			↑*Fusobacteria*
			↑*Fusobacteriaceae*
Schwimmer et al. (2019)	87 biopsy-proven NAFLD children (age range, 8–17 years) patients and 37 children with obesity without NAFLD (controls) (United States of America)	Fecal microbiome:16S ribosomal RNA amplicon sequencing and metagenomic shotgun sequencing	Patients with severe fibrosis: ↑*Prevotella copri*
Ponziani et al. (2019)	21 patients diagnosed with NAFLD-related cirrhosis and HCC, 20 patients with NAFLD-related cirrhosis without HCC, and 20 controls (Italy)	Fecal microbiome: 16S rRNA gene sequencing	Patients with cirrhosis
			↑*Enterobacteriaceae*
			↑*Streptococcus*
			↓*Akkermansia*
			Patients with HCC
			↑*Bacteroides*
			↑*Ruminococcaceae*
			↓*Bifidobacterium*
Lee et al. (2020)	171 biopsy-proven NAFLD patients and 31 non-NAFLD controls (Korea)	Fecal microbiome: 16S rRNA sequencing	Non-obese patients with progressing fibrosis:↓*Ruminococcaceae*
			↑*Veillonellaceae*
Tsai et al. (2020)	50 biopsy-proven NAFLD patients and 25 biopsy-proven non-NAFLD controls (China)	Fecal microbiome: 16S ribosomal RNA gene sequencing	Patients with NAFLD
			↑*Bacteroidetes*
			↓*Firmicutes*
			Patients with NAFLD or NASH
			↓*Ruminococcaceae UCG-010*
			↓family *Ruminococcaceae*
			↓order *Clostridiales*
			↓class *Clostridia*
Yu et al. (2021)	23 NAFLD patients with elevated liver enzymes and 22 healthy controls (Korea)	Fecal microbiome: 16S rRNA sequencing	Patients with NAFLD: ↑F/B
			↑*Veillonella*
			↓*Oscillospiraceae_UCG-003*
			↑*Collinsella*
			↓*Ruminococcus*
			↑*Latilactobacillus*
			↓*Prevotella-9*
			↑*Dialister*
			↓*Lachnospiraceae_UCG-004*
			↑*Bifidobacterium*
			↓*Lachnospiraceae_UCG-003*
			↓*Lachnospiraceae_UCG-010*
			↓*Erysipelotrichaceae_UCG-003*
Leung et al. (2022)	90 ultrasound-diagnosed NAFLD patients and 90 controls (China)	Fecal microbiome: shotgun metagenomic sequencing	Patients with NAFLD
			↓*Methanobrevibacter*
			↑*Dorea formicigenerans*
			Patients with advanced fibrosis
			↑*Slackia*
Demir et al. (2022)	78 patients with NAFLD and 16 controls (Germany)	Fecal microbiome: fungal internal transcribed spacer 2 sequencing	Patients with NASH and F2-F4 fibrosis
			↑log-ratio for *Mucor* sp.*/Saccharomyces cerevisiae*
			↑log-ratio for *Candida albicans/Saccharomyces cerevisiae*

Abbreviations: NAFLD, non-alcoholic fatty liver disease; NASH, non-alcoholic steatohepatitis; F, fibrosis; F/B, *Firmicutes/Bacteroidetes;* HCC, hepatocellular carcinoma.

## 3 Influence of UDCA on the gut microbiome in NAFLD

### 3.1 UDCA biosynthesis

Bile acids, amphipathic biological detergents, serve a wide range of regulatory functions in humans. PBAs, mainly including chenodeoxycholic acid (CDCA) and cholic acid (CA), are *de novo* synthesized in the liver by regulation of two main enzymes, sterol 27-hydroxylase (Cyp27a1) and cholesterol 7 alpha-hydroxylase (Cyp7a1). After being initially synthesized from cholesterol in the liver, bile acids undergo biotransformation to SBAs, secreted into bile, conjugated with either taurine or glycine, and then deconjugated and dehydrogenized by the microbiome residing in the gut. UDCA is produced as a collaborative effort by the production of primary bile acid in the host and the gut microbiome. The predominant secondary bile acids include DCA and lithocholic acid, formed from CA and CDCA, respectively (Sinha et al., 2020). Found in small quantities in the total bile acid (BA) pool, UDCA is a kind of SBA with beneficial effects on humans due to its hydrophilicity. In humans, the conversion of chenodeoxycholic acid to UDCA is facilitated by 7α- and 7β-hydroxysteroid dehydrogenases through epimerization of the 7-hydroxy group. Furthermore, this biotransformation of UDCA has been applied in artificial large-scale production and could be applied with a rational design of the bacterial consortium (Zhang et al., 2019; Zhou et al., 2023). Specific gut microbiomes are also essential in UDCA biosynthesis. There is a positive correlation between UDCA and the gut microbiome with aforementioned biocatalysts, including *Ruminococcus*, Peptococcaceae, *Roseburia*, and *Faecalibacterium prausnitzii* (Dempsey et al., 2019; Huang et al., 2019; Pearson et al., 2019).

### 3.2 Changed gut microbiome profile modulated by UDCA

**FIGURE 2:**
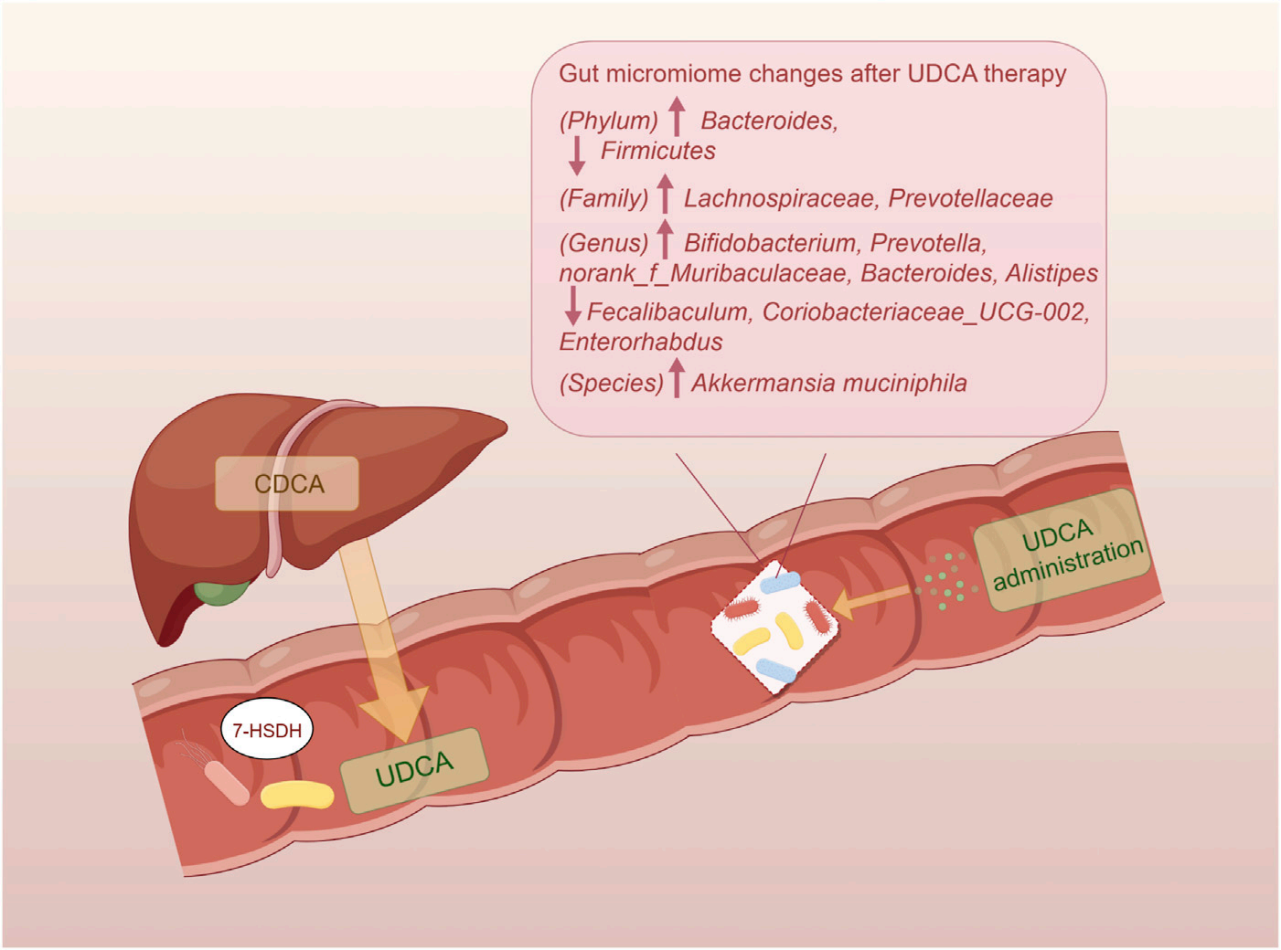
Impact of UDCA on the gut microbiome. The endogenous UDCA is produced by the gut microbiome with their 7-HSDH, which converts CDCA into UDCA. The administration of UDCA has the ability to regulate the intestinal bacteria and restore homeostasis. UDCA, ursodeoxycholic acid; 7-HSDH, 7-hydroxysteroid dehydrogenase; CDCA, chenodeoxycholic acid.

UDCA and its conjugated forms have been proven to be effective regulators of the gut microbial community structure (Van den Bossche et al., 2017; Tang et al., 2018; Pearson et al., 2019) (Figure 2). In the colitis mouse model, tauroursodeoxycholic acid (TUDCA) and UDCA could normalize the *Firmicutes/Bacteroidetes* ratio and elevate the abundance of *Akkermansia* and *Prevotellaceae* (Van den Bossche et al., 2017). UDCA therapy also resulted in attenuation of pathogenesis of infectious intestinal disorders, like *Clostridioides difficile* infection, due to modulation in colonization resistance against pathogenic bacteria and immune response (Winston et al., 2020). Recent studies highlighted the role of *Lachnospiraceae* family in regulating microbial community structure through UDCA, a beneficial taxon known to participate in the production of SCFAs and conversion of SBAs (Sorbara et al., 2020; He et al., 2022). With elevated oral gavage of UDCA, the abundance of *Lachnospiraceae* in the rodent intestine increased in a dose-dependent manner (Wilson et al., 2021). Furthermore, the relative abundance of *Lachnospiraceae* remained significantly positively correlated with fecal levels of conjugated forms of UDCA (Huang et al., 2019; Kimmel et al., 2022) when treating with a prevalent traditional Chinese medicine named *Gracilaria lemaneiformis*. Additionally, genera *Bifidobacterium* and *Prevotella* were also reported to be related to elevated UDCA (Ghaffarzadegan et al., 2019). As facilitators and maintainers of human intestinal health, *Alistipes* was known to produce acetate to suppress the inflammatory response and tended to increase after UDCA treatment in NAFLD (Li et al., 2021). However, the relative abundance of the *Faecalibaculum* genus, that was generally considered beneficial, tended to be elevated in NASH mice and decreased with UDCA treatment (Li et al., 2021; He et al., 2022). Totally, numerous studies demonstrated an increasing trend for a beneficial microbiome with UDCA therapy, whereas a few studies exhibited the opposite results. The potential putative cause is that specific bacterial taxa play a disparate role in the pathogenesis of chronic liver diseases.

A novel concept of the gut microbiome–UDCA-host axis has been proposed in latest research studies, elucidating one of the mechanisms by which the microbiome modulates host metabolisms. The gut microbiome occupies a significant position in the synthesis of UDCA. UDCA subsequently alters the bile acid pool through affecting BA-producing bacteria and changes farnesoid X receptor (FXR) and Takeda G protein-coupled receptor 5 (TGR5) signaling ways in the host. However, the difference in affinity and agonist ability toward FXR and TGR5 of different bile acids complicates the axis, such that UDCA has no ability to activate FXR but is an agonist for TGR5, whereas TUDCA activates both of these receptors (Wilson et al., 2021). According to this axis, UDCA treatment results in alterations in the levels of bacteria associated with bile acid metabolism, ultimately leading to a reduction in unconjugated BAs and an increase in conjugated BAs (Chen et al., 2020).

## 4 Possible mechanisms of UDCA in NAFLD

**FIGURE 3:**
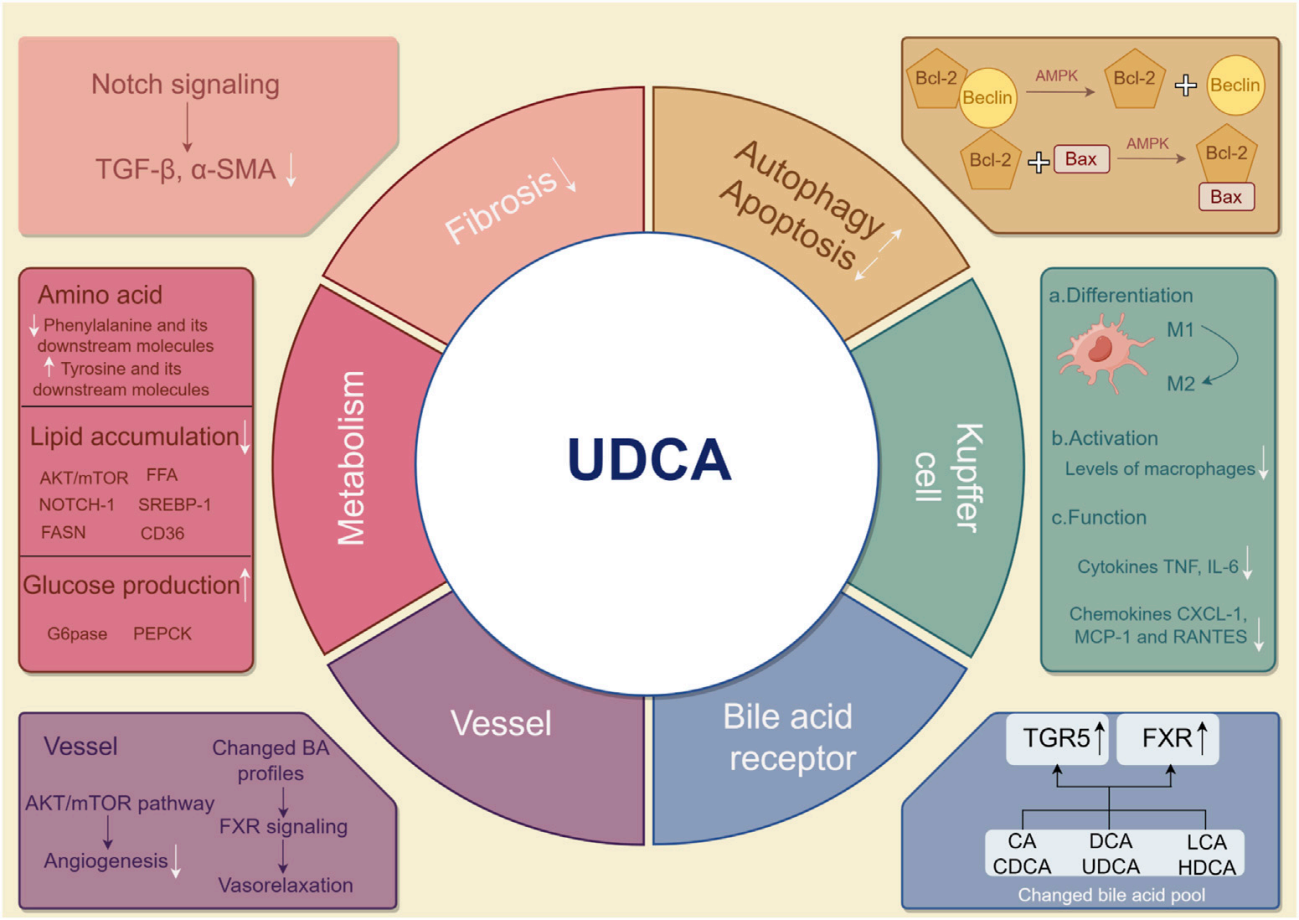
Possible mechanisms by which UDCA mitigates NAFLD. UDCA may treat NAFLD in the following ways. In hepatic cells, induced autophagy and alleviated apoptosis are found after UDCA therapy. Fibrosis and major metabolisms can be effectively modulated by UDCA. In Kupffer cells in the liver, UDCA attenuates the pro-inflammatory response. Angiogenesis is diminished, and vasorelaxation is found in vessels around the liver. By modulating the bile acid pool, UDCA indirectly and directly influences TGR5 and FXR. UDCA, ursodeoxycholic acid; NAFLD, non-alcoholic fatty liver disease; UDCA, ursodeoxycholic acid; TGR5, Takeda G protein-coupled receptor 5; FXR, farnesoid X receptor.

Clinical studies comparing NAFLD and controls reported higher levels of total serum bile acids, secondary bile acids, deoxycholic acids, and chenodeoxycholyl-conjugates (Jiao et al., 2018; Caussy et al., 2019), while the relative abundance of unconjugated acids and UDCA tended to be decreased (Tang et al., 2019). UDCA, with hepatoprotective effects, is a gut microbiome-producing metabolite that significantly contributes to treating primary biliary cholangitis; however, it currently remains unknown if and how UDCA therapy confers protection against NAFLD. There are several possible mechanisms underlying the protective effect of UDCA on liver function, as shown in Figure 3. Although some of them have not been directly evidenced in NAFLD, it provides comprehensive possibilities for UDCA to modulate host metabolism and benefit liver functions.

### 4.1 Modulation in host metabolism (amino acid, lipid, and glucose)

Amino acid metabolism mainly occurs in the liver, and metabolome analysis can be potentially applied in characterizing NAFLD. Patients generally exhibited liver dysfunction accompanied by instability of amino acid profiles. The ratio of branched-chain amino acids (BCAA) to aromatic amino acids (AAA) has been known as a diagnostic biomarker for assessing the severity of liver diseases (Middendorf et al., 2019; Devriendt et al., 2021). A quantitative model with superior predictive power was constructed base on amino acid profiles to identify NASH patients (Yamakado et al., 2017). Unlike antioxidants vitamins E and C, improving the amino acid metabolism dysfunction is a unique mechanism of UDCA in relieving hepatic steatosis. The metabolomic pathway of biosynthesis of phenylalanine, tyrosine, and tryptophan, was filtered out after UDCA administration in patients with choledocholithiasis (Guan et al., 2022). In addition, the level of L-phenylalanine and its downstream molecules was significantly decreased, while the level of N-acetyltryptophan, a tryptophan metabolite, increased with UDCA treatment in patients with liver dysfunction (Kim et al., 2018). In livers of mice, FXR activation could be a choice to modulate amino acid degradation via Hal and Prodh and ureagenesis via Cps1, Ass1, and Arg1 (Massafra et al., 2017). Further studies involving how UDCA influences amino acid levels in hepatic steatosis are required.

Hepatic lipid dysfunction serves as one of the most important mechanisms in NAFLD. The inhibitory effect of UDCA on adipogenic genes was confirmed, especially on the sterol-regulatory element-binding protein-1 (Srebp-1) family and its downstream enzymes such as Fasn and CD36. As a key molecule in adipogenesis and fat accumulation in the liver, Srebp-1 was upregulated in NAFLD according to most studies and downregulated upon treating with UDCA and its derivative norursodeoxycholic acid (norUDCA) (Chen et al., 2021; Marchiano et al., 2022). The downregulation of Srebp-1 was mediated by various pathways, such as the AKT/mTOR/Srebp-1 pathway. Activated by AKT, mTOR upregulated the CRTC2 complex and then promoted the activity of Srebp-1 (Hu et al., 2019). UDCA also repressed Srebp-1 via free fatty acid receptor 4 (FFA4)-dependent and Notch1 signaling transduction pathways (Xu et al., 2022). In addition to Srebp-1, Notch1 signaling repression simultaneously inhibited the expression of CD36 (Gheibi et al., 2019). CD36, namely, fatty acid translocase, formed a complex with insulin-induced gene-2 (INSIG-2) and activated Srebp-1 and adipogenesis (Zeng et al., 2022). However, the direct association between CD36 and UDCA remains unclear.

As a metabolic disease involving multiple systems, NAFLD is closely related to insulin resistance and dysregulated glucose homeostasis. UDCA significantly diminished gluconeogenesis and Notch1 signaling in the liver, evidenced by reduced protein levels and mRNA expression of glucose-6-phosphatase (G6pase) and PEPCK in leptin deficiency obese mice (Chen et al., 2019). Nevertheless, no significant changes were observed in levels of gluconeogenic genes after UDCA administration (Marchiano et al., 2022). Moreover, molecules related to cellular apoptosis were also found to be involved in glucose metabolism. The pro-apoptosis biomarker p53 weakens the pentose phosphate pathway glucose flux and intracytoplasmic carbohydrate storage (Jiang et al., 2011). Similarly, miR34a in the apoptosis pathway also dephosphorylates HMG reductase, influencing glucose biosynthesis (Castro et al., 2013). Both miR34a and p53 could be regulated by UDCA, indicating its potential mechanisms in glucose metabolism.

### 4.2 Restoration of apoptosis and oxidative stress

In NAFLD, autophagy is impaired while apoptosis increases, and UDCA treatment can reverse this alteration (Panzitt et al., 2021). The binding of Bcl-2 to Beclin inhibited autophagy induced by Beclin while maintaining the anti-apoptosis function of Bcl-2 at a high level. Hence, disrupting the Bcl-2–Beclin complex generally prevented premature aging (Fernandez et al., 2018; Papini et al., 2023). Unlike the Bcl-2–Beclin complex, the Bcl-2–Bax complex is proven to be highly effective in preventing apoptosis *in vitro* (Lu et al., 2017). UDCA exerted a favorable influence on the liver via apoptosis suppression and autophagy enhancement by promoting dissociation of the Bcl-2/Beclin complex and inhibiting dissociation of the Bcl-2/Bax complex through AMP-associated protein kinase activation (Wu et al., 2020). UDCA suppressed a pro-apoptosis pathway of microRNA-34a/Sirtuin 1/p53 and subsequent cellular apoptosis in the rat liver and primary rat hepatocytes (Castro et al., 2013). However, UDCA was reported to only reduce miR-34a in the vesicle-free fraction of serum and did not have a similar effect on the liver. Additionally, the potential of UDCA in enhancing the expression of the endoplasmic reticulum (ER) stress markers CHOP and Gpr78 and inducing apoptosis was also reported (Mueller et al., 2018). The following may explain for this contradiction: the FXR inhibition led by UDCA resulted in high cholesterol storage in the liver and the initiation of unfolding protein response, which was an attempt to restore the ER homeostasis but finally a promotor of apoptosis. Notably, while UDCA induces endoplasmic reticulum stress, apoptotic indicators like caspase-3 had no significant changes since apoptotic threshold and cytoprotective ability were enhanced (Mueller et al., 2018; Ali et al., 2020).

Reactive oxygen species (ROS), mainly induced when an electron escapes during ATP synthesis, causes an imbalance between oxidants and antioxidants, leading to mitochondrial dysfunction, excessive β-oxidation, and oxidative stress. ROS inflicts fatal damage to hepatocytes and also activates the inflammation of hepatic stellate cells, which further promoted the progress from NAFLD to liver cirrhosis (Borrelli et al., 2018; Shum et al., 2021). Furthermore, the superior clinical effect of antioxidants *versus* UDCA in NAFLD has been determined, including vitamins E and C (Borrelli et al., 2018; Fouda et al., 2021). Administration of UDCA notably altered the bile acid profile and subsequent hepatic steatosis. Bile acids with amphiphilicity modulated the activity of the electron transfer chain, membrane permeability, and biological synthesis to improve mitochondrial functions. UDCA therapy was revealed to improve hepatocyte mitochondrial function in rodent models (Chen et al., 2019; Pérez et al., 2021). Despite preclinical research studies, clinical evidence that UDCA depends on the mitochondria to treat NAFLD was still lacking. The combinational therapy of UDCA and antioxidants exhibited a remarkable effect with improved malondialdehyde and glutathione for NAFLD in animal experiments (Gheibi et al., 2019). Paradoxically, a few recent clinical studies failed to prove the capability of UDCA to change the oxidative status, and more studies are necessary to be designed to affirm the true role played by UDCA in oxidative stress.

### 4.3 Vasorelaxation and angiogenesis inhibition

The progression of hepatic steatosis to cirrhosis resulted in increased portal blood flow and splanchnic vasodilation due to enhanced circulating endogenous vasodilators and inhibited response to vasoconstrictors. Flow-mediated dilatation notably decreased in the brachial artery in NASH patients, indicating impaired endothelial function (Al-Hamoudi et al., 2020). Finally, the portal hypertension emerged as a consequence of the abovementioned process and increased intrahepatic vascular resistance (Chalasani et al., 2020; Sauerbruch et al., 2021). Bile acid, especially UDCA, is a crucial modulator in enterohepatic circulation. Elevated levels of hepatic angiogenesis markers were induced by liver inflammation and oxidative stress, while UDCA showed the ability to attenuate dysbiosis (Chen et al., 2019). Moreover, human M1 macrophages, the secretor of angiogenic stimulator VEGF, is inhibited in NAFLD and restored with administration of UDCA (Li et al., 2018; Chen et al., 2019). Hydrophilic UDCA had no direct evidence of a vasoactive effect but influenced angiogenesis (Jung and Hwang, 2021); however, UDCA might affect the vascular tone by altering bile acid profiles.

### 4.4 Regulation of inflammatory signaling pathways and liver fibrosis

Kupffer cells (KCs) are intrahepatic macrophages that represent the core immune cells during the pathogenesis of NAFLD. UDCA could modulate KCs and alleviate hepatic pathology via three ways: 1) differentiation: two subtypes of macrophages M1 and M2 represented pro-inflammatory and anti-inflammatory response, respectively. UDCA activated macrophage M2 (Chalasani et al., 2020) and modulated polarization between M1 and M2 via Notch1 signaling (Chen et al., 2019). 2) Activation: UDCA treatment resulted in lower levels of activated macrophages marked by soluble CD163 (Bossen et al., 2023). 3) Function: macrophage functions modulated by UDCA via secreting cytokines TNF and IL-6, as well as chemokines CXCL-1, MCP-1, and RANTES (Ludwig et al., 2018; Labiano et al., 2022; Marchiano et al., 2022). Interestingly, UDCA–lysophosphatidylethanolamide contributed to lower pro-inflammatory TNF and MCP-1 via KCs. It also activated the PI3K/AKT pathway to compensate for depressed hepatocyte proliferation due to TNF decline (Ludwig et al., 2018).

Signals including transforming growth factor-β (TGF-β), osteopontin, α-smooth muscle actin (α-SMA), and TAZ suggested the activation of the hepatic stellate cells (HSCs) (Kuchay et al., 2020; Dong et al., 2021). Bone morphogenetic protein 8B, found in TGF-β/BMP superfamily and absent in healthy livers, induced the proinflammatory phenotype of HSCs (Vacca et al., 2020). A recent research indicated the ability of UDCA to degrade TGF-β and of further enhancing antitumor immunity (Shen et al., 2022). Treating a rodent model of hepatic steatosis with UDCA and norUDCA significantly reduced fibrosis biomarkers TGF-β and α-SMA at the genetic level (Marchiano et al., 2022). Downregulation of Notch signaling by UDCA resulted in release of the signals that participated in the activation of resident HSCs synchronously (Zhu et al., 2018). Furthermore, liver regeneration significantly contributes to resistance of fibrosis, resulting in decreased macrophage infiltration and collagen deposition, which is promoted by UDCA via inhibitor of the DNA binding 1-dependent pathway (Dong et al., 2021). Even if potential protection of UDCA against hepatic fibrosis is nearly confirmed, the optimal dose and duration needs further exploration before becoming one of the first-line anti-fibrotic therapies for NAFLD (Ratziu et al., 2011; Parikh et al., 2016; Nadinskaia et al., 2021).

As a liver health promoter, UDCA targets various mechanisms corresponding with the pathophysiological process of NAFLD and NASH, incorporating effect of the glucose, lipid, and amino acid metabolism; cellular-level apoptosis; autophagy and oxidative stress; liver tissue-level blood vessels and inflammatory responses; and fibrosis. The effect of UDCA has been determined clearly in animal and cell experiments; however, the real clinical application needs explorations in future.

### 4.5 Bile acid receptors

FXR is widely expressed in the liver and ileum, functioning as the regulator of bile acid, lipid, and glucose metabolism. Clinical and preclinical studies confirmed that activators of FXR, OCA, and DCA showed a protective effect against liver steatosis (Younossi et al., 2019; Huang et al., 2021; Gillard et al., 2022; Rinella et al., 2022; Zhuge et al., 2023). Nevertheless, there is a consensus that UDCA is a weak ligand with little activation of FXR in humans, and it even exerts FXR-antagonistic effects (Li et al., 2021; Marchiano et al., 2022; Brevini et al., 2023; Wang et al., 2023). In current studies, UDCA is found to potentially interfere in the FXR pathway indirectly, mainly via gut microbiome remodeling and bile acid profile alteration. Patients receiving FXR-antagonistic UDCA surprisingly exhibited an activated effect of FXR with increased FGF19 (a FXR target gene), majorly due to the remodeled gut microbiome and induced high bile acid-deconjugating enzymes by UDCA. It subsequently enabled secondary modification and higher activity for endogenous FXR agonists (Ovadia et al., 2020). Without a notable activation effect on FXR *in vitro*, UDCA restored the expression and transduction of the FXR pathway *in vivo* with lower expression of Cyp7a1 (Marchiano et al., 2022). The efficacy of UDCA was contingent upon the presence of endogenous FXR ligands, as evidenced by its failure to increase FGF15 (a FXR target gene) in animals with biliary obstruction (Zaufel et al., 2021). It suggested that FXR-agonistic bile acids played a contributory role in mechanisms of UDCA. The evidence also suggested a possible role of reduced β-muricholic acids (MCAs), another FXR antagonist, in the reduction of hepatic lipid contents by UDCA, which could not be explained in humans in the absence of MCA (Fujita et al., 2017).

TGR5, also known as GPBAR1, is widely distributed in the skeletal muscle, white and brown adipocytes, ileum, and entero-hepatic tissues except hepatic parenchymal cells. TGR5-dependent pathways are crucial for protecting hepatocytes from injuries, involving improvement of glucose homeostasis, gallbladder dilatation, hepatic inflammation, and energy expenditure (Carino et al., 2017; Ginos et al., 2018; Iracheta-Vellve et al., 2018; Bidault-Jourdainne et al., 2021; Wang et al., 2022). In addition, diminished TGR5 signals were found to be correlated with downregulated secondary bile acids, attributed to alterations in the abundance of bacteria involved in bile acid transformation (Spatz et al., 2021). UDCA possessed the potential to improve histology for NASH and NAFLD as a treatment with TGR5 activation (Carino et al., 2017; Finn et al., 2019). The activated pathway of TGR5 signaling by UDCA has been certified to attenuate primary sclerosing cholangitis (PSC) and inhibit the proliferation of colorectal cancer cells and *Escherichia coli* infection (Reich et al., 2021; Zhang et al., 2021; He et al., 2022). However, the evidence on mechanisms of UDCA treatment for NAFLD via the TGR5 pathway is limited, which is possibly ascribed to the less abundance of TGR5 on hepatic parenchymal cells. One of possible mechanisms was stimulation of GLP-1 (a TGR5 target gene) release from intestinal L cells to protect hepatocytes against the inflammatory response (Carino et al., 2019; Marchiano et al., 2022). In addition to GLP-1, mechanisms of the UDCA-inducing TGR5 pathway need more explorations of the involved molecules to better illustrate the effect brought about by this pathway.

## 5 Therapeutic target of the UDCA–gut microbiome axis for NAFLD

### 5.1 Evidence on UDCA application in NAFLD

In previous clinical trials, UDCA administration had a beneficial effect on NAFLD and NASH patients with effectively improved liver function biomarkers (Dufour et al., 2006; Leuschner et al., 2010; Ratziu et al., 2011; Nadinskaia et al., 2021). Even the efficacy and safety of high-dose UDCA were already certified. However, significant differences in liver histology could not be detected using either invasive liver fibrosis tests or a second biopsy in humans, which was inconsistent with animal studies. In a rodent model of NASH, UDCA significantly attenuated hepatic inflammation histologically (Li et al., 2021). Since no evidence of significant histological improvement in large-scale clinical trials was demonstrated presently, UDCA could only be used as an adjunct to attenuate NAFLD and prevent it from progressing to severe fibrosis.

Interestingly, UDCA could partially restore intestinal dysbiosis induced by NAFLD and repair gut barrier integrity with increased expression of claudin-1 and ZO-1. At the phylum level, NAFLD mice treated with UDCA exhibited a decreased relative abundance of *Firmicutes* and increased relative abundance of *Bacteroidetes*. At the genus level, NAFLD mice treated by UDCA exhibited a lower abundance of *Fecalibaculum*, *Coriobacteriaceae_UCG-002*, and *Enterorhabdus* and higher abundance of *norank_f_Muribaculaceae*, *Bacteroides*, and *Alistipes* (Li et al., 2021). TUDCA, a conjugated bile acid derivative undergoing higher hydrophilicity as a candidate drug, attenuated hepatic steatosis and inflammation in the NAFLD mouse model (Wang et al., 2018). In the TUDCA-treated group, the changes in *Proteobacteria*, *Paraprevotella*, and *Dehalobacterium* tended to be partially reversed (Wang et al., 2018). Hence, these TUDCA-regulating microbiomes in the animal intestine might mediate the improvement of hepatic steatosis by gut–liver crosstalk. A side chain-shortened homolog of UDCA, norUDCA, also resulted in amelioration of NAFLD with improved liver function, yet without evidence that norUDCA remodeled the intestine microbiome (Traussnigg et al., 2019; Marchiano et al., 2022).

### 5.2 Dietary therapy

Currently, a balanced diet and healthy lifestyle are considered the best strategies for NAFLD patients. Notably, the Japanese diet pattern and Mediterranean diet were found to be effective in lowering the severity of liver fat accumulation and fibrosis in clinical studies (Montemayor et al., 2022; Matsumoto et al., 2023). These two diet patterns are characterized by a high intake of soybeans and soybean foods, vegetables, fruits, and seafood. In dietary therapy for hepatic steatosis, it was commonly observed that specific food remodeled the intestine microbial community. Changes in the bacteria profile led to comprehensive biochemical activities represented by alterations in bile acid metabolism, ultimately improving hepatic steatosis and altering the relative abundance of UDCA. *Gracilaria lemaneiformis* (GLP) increased the abundance of UDCA and TUDCA via elevating the abundance of *Lachnospiraceae_NK4A136_group* and *Roseburia* in the mouse intestine, which possibly explained for GLP protecting the liver from damage caused by a high-fat diet (Huang et al., 2019). Therefore, GLP could be used as a functional food to diminish NAFLD. Apple polyphenol extract (APE) significantly reduces the relative abundance of *Lactobacillus* and increases the relative abundance of *Akkermansia*, leading to reduced fecal UDCA in the NAFLD mouse model (Li et al., 2021). Interestingly, in another study, enriched *Lactobacillus* in both the grass carp and chicken groups was positively related to UDCA (Li et al., 2022). Collectively, increased UDCA tended to cause liver homeostasis in spite of different abundances of *Lactobacillus*. Meanwhile, more cautions should be taken in the prevention of NAFLD by dietary therapy, involving GLP, APE, and dietary white meat, before the efficiency is confirmed after robust clinical trials.

### 5.3 Probiotics and prebiotics

Nutritional interventions have been the first approach toward a healthy lifestyle as the main strategy to manage NAFLD. The synbiotic led to a significant steatosis remission in an *in vivo* rat model (Juarez-Fernandez et al., 2021). A variety of synbiotics and probiotics were suggested to be effective in changing gut dysbiosis in order to have a beneficial effect on NAFLD, as observed in numerous clinical studies (Ahn et al., 2019; Behrouz et al., 2020; Scorletti et al., 2020; Mohamad Nor et al., 2021) and rodent experiments (Wang et al., 2020). In animal studies, the intervention with the synbiotic of *Akkermansia muciniphila* and quercetin combination resulted in improvement of steatosis. This therapeutic capacity was shown to be driven by increased levels of hydrophilic bile acid UDCA along with the altered gut microbiome, in which a higher abundance of *Cyanobacteria* and *Oscillospira* as well as lower levels of *Actinobacteria*, *Lactococcus*, *Lactobacillus*, and *Roseburia* were observed (Juarez-Fernandez et al., 2021). Further investigations regarding the mechanisms of gut microbiome modulation and the shift in UDCA underlying beneficial effects of synbiotics and probiotics need to be conducted in clinical patients.

### 5.4 Fecal microbiota transplantation

Increasing evidence indicated that FMT is a novel approach for improving the manifestations of NAFLD by reconstructing the intestinal microecological balance and diversity. A randomized control trial reported that FMT has the potential to reduce gut permeability in NAFLD patients (Craven et al., 2020). Another clinical trial demonstrated that FMT affected hepatic DNA methylation and levels of phenylacetate- and choline-derived metabolites in individuals with NAFLD. As for gut microbial composition changes, *Blautia wexlerae*, a potential anti-obesogenic probiotic, was increased upon allogenic FMT compared with autologous FMT (Stols-Gonçalves et al., 2023). The proportions of beneficial bacteria *Bacteroidetes*, *Christensenellaceae*, and *Lactobacillus* were also increased by FMT intervention. Contrastingly, the proportions of *Escherichia–Shigella*, *Odoribacter*, and *Oscillibacter* decreased in patients with NAFLD after FMT (Zhou et al., 2017; Xue et al., 2022). These results showed that NAFLD-associated gut microbiome disturbance was, at least partially, corrected after FMT. While changes in the microbial metabolites, such as butyrate concentrations of the cecal content (Zhou et al., 2017), plasma phenylacetylcarnitine, and phenylacetylglutamine (Stols-Gonçalves et al., 2023), as well as gut microbiome structure were indicated, it would be highly interesting to explore the correlation between another microbial metabolite, bile acids, especially UDCA, and FMT intervention in NAFLD.

## 6 Discussion

Although NAFLD has become a pandemic attributed mainly to Western diet, the current methods of treatment are still limited. Accumulating evidence has recently shown the interactions between bile acids and gut microbiome and their roles in NAFLD. Additionally, a growing number of studies have indicated that certain bile acids, such as UDCA, exhibit beneficial effects on NAFLD through multiple mechanisms. In this study, the possible functional roles of UDCA and the gut microbiome in NAFLD were discussed.

However, there are still many limitations in this regard regarding existing research. First, there are many factors that affect the microbial community, and different research studies yielded inconsistent conclusions with different detection and modeling methods. Second, the significant therapeutic efficacy of UDCA has been clearly confirmed in animal models, whereas the improvement of liver biochemistry, not histological changes, is recognized in clinical patients. The reason for this inconsistency deserves further study and discussion so as to clarify the actual effect of UDCA in the treatment of NAFLD. Third, extensive animal experiments were analyzed when elucidating the interplay between the NAFLD, UDCA, and gut microbiome. It is well-recognized that lifestyle, including eating habits and exercise, is closely related to the progression of NAFLD. However, there are significant differences in lifestyle between humans and animals, hindering the representativeness of animal experimental results. Furthermore, because the bile acid composition in animals is substantially different from that in humans, changes in bile acid pool and host metabolism in humans and animal models of NAFLD must be interpreted cautiously. For instance, muricholic acid in the bile acid pool of mice is almost undetectable in humans.

Hence, the evidence from current studies is inadequate. The recovery of bacterial function may be an important direction of treatment of NAFLD, including further research on direct UDCA application, dietary therapy, probiotics, and standardization of FMT. In the future, more clinical and animal studies are necessary to explore the clinical efficacy and specific mechanisms between UCDA and the gut microbiome in NAFLD.

## Figures and Tables

**Table:** 

**NAFLD**	non-alcoholic fatty liver disease
**NASH**	non-alcoholic steatohepatitis
**PNPLA3**	patatin-like phospholipase domain-containing 3
**TM6SF2**	transmembrane 6 superfamily member 2 protein
**MBOAT7**	membrane-bound O-acyltransferase domain-containing 7
**LPL**	lipoprotein lipase
**FMT**	fecal microbiota transplantation
**PBAs**	primary bile acids
**SBAs**	secondary bile acids
**UDCA**	ursodeoxycholic acid
**G-**	Gram-negative
**F/B**	*Firmicutes/Bacteroidetes*
**DCA**	deoxycholic acid
**CDCA**	chenodeoxycholic acid
**CA**	cholic acid
**Cyp27a1**	sterol 27-hydroxylase
**Cyp7a1**	cholesterol 7 alpha-hydroxylase
**BA**	bile acid
**TUDCA**	tauroursodeoxycholic acid
**FXR**	farnesoid X receptor
**TGR5**	Takeda G protein-coupled receptor 5
**BCAA**	branched-chain amino acid
**AAA**	aromatic amino acid
**Srebp-1**	sterol-regulatory element binding protein-1
**norUDCA**	norursodeoxycholic acid
**FFA4**	free fatty acid receptor 4
**INSIG-2**	insulin-induced gene-2
**ER**	endoplasmic reticulum
**ROS**	reactive oxygen species
**KCs**	Kupffer cells
**TGF-β**	transforming growth factor-β
**α-SMA**	α-smooth muscle actin
**HSC**	hepatic stellate cell
**MCA**	β-muricholic acids
**PSC**	primary sclerosing cholangitis
**GLP**	*Gracilaria lemaneiformis*
**APE**	apple polyphenol extract
